# Longitudinal measurements of neutrophil-to-lymphocyte ratio in nasopharyngeal cancer treated with concurrent chemoradiotherapy

**DOI:** 10.1371/journal.pone.0292591

**Published:** 2023-10-09

**Authors:** Pooriwat Muangwong, Nontiya Homkham, Wattanapong Narueban, Chin Tadadoltip, Chayaporn Jongjumnien, Nuttida Taenawakun, Jutamas Teerapattanaphong, Imjai Chitapanarux

**Affiliations:** 1 Faculty of Medicine, Chiang Mai University, Chiang Mai, Thailand; 2 Northern Thai Research Group of Radiation Oncology (NTRG-RO), Faculty of Medicine, Chiang Mai University, Chiang Mai, Thailand; 3 Faculty of Public Health, Thammasat University, Bangkok, Thailand; Abu Dhabi University, UNITED ARAB EMIRATES

## Abstract

**Objective:**

We study factors affecting neutrophil-to-lymphocyte ratio (NLR) and its changes throughout the treatment (ΔNLR) of nasopharyngeal carcinoma (NPC) underwent chemoradiotherapy (CCRT) followed by adjuvant chemotherapy (AC) and oncological outcomes including overall survival (OS) and disease-free survival (DFS).

**Methods:**

Data from 81 NPC patients was retrospectively evaluated. NLRs were obtained from first week of CCRT (pre-CCRT), last week of CCRT (end-CCRT), and at last cycle of AC (end-AC). Pre-CCRT NLR was categorized into “low” and “high”. End-CCRT and end-AC ΔNLRs were divided into “increased” and “decreased” based on NLR at these two timepoints relative to the value at pre-CCRT. Associations between sex, age, cancer stage and NLR, ΔNLRs were investigated. OS and DFS were reported.

**Results:**

Median NLR at pre-CCRT (2.47) was lower than NLR at end-CCRT (6.29) and end-AC (3.77) (*P*-value = 0.043). Advanced cancer stage associated with high pre-CCRT NLR (*P*-value = 0.047). Male gender was associated with "increased" end-CCRT ΔNLR, whereas male gender and age ≤51 were associated with "increased" end-AC ΔNLR. Three-year OS and DFS rates were 85.25% and 76.39%, respectively. There were no statistically significant differences observed in OS and DFS among groups categorized by pre-CCRT NLR, ΔNLRs, gender, age, and cancer stage.

**Conclusions:**

NLR increases during NPC treatment. Advanced staging is associated with higher baseline NLR. Increased ΔNLR is associated with male gender at end-CCRT and male gender with age ≤51 years at end-AC. No relation between NLR and its dynamic change with either OS or DFS was demonstrated.

## Introduction

Nasopharyngeal carcinoma (NPC) develops from epithelial lining of nasopharynx. The disease is more prevalent in East and Southeast Asia which accounts for approximately 70% of the incidence worldwide [1]. Due to the nature of the disease, which typically spreads through the lymphatic system, it commonly presents in locally advanced stage that accumulates about 92% of non-metastatic disease and the treatment is concurrent chemoradiotherapy (CCRT) with or without either induction or adjuvant chemotherapy (AC) [2, 3].

Neutrophil-to-lymphocyte ratio (NLR) is one of the prognostic markers of cancer. High NLR before treatment associates with poor overall survival (OS) in many cancers [4–6]. The change of post-treatment NLR relative to initial NLR (ΔNLR) also represents the prognosis. Increased ΔNLR in various treatment modalities demonstrates inferior treatment outcomes in many cancers including biliary tract cancer [7, 8], colorectal cancer [9], breast cancer [10, 11], and glioblastoma [12].

In NPC, elevated pre-treatment NLR likewise links to poor OS [13, 14]. It also associated with higher cancer stage and greater number of cervical lymph node metastasis [15]. High post-CCRT NLR correlates with poor OS [16]. High ΔNLR of post-CCRT vs. pre-CCRT also diminishes OS. NLR also increases throughout the course of radiotherapy (RT) [9] because of inflammation of irradiated tissue [17, 18].

In this study, we investigated NLR values and ΔNLR throughout CCRT and AC as well as the association between NLR and ΔNLR and survival rate in NPC.

## Materials and methods

We retrospectively obtained data from the entire group of stage II–IVA non-metastatic NPC patients who were treated in the CCRT and AC arms of the prospective cohort study conducted between 2015 and 2018 and enrolled among five cancer centers in Thailand [19]. In the prospective study, patients were stratified by center and randomized 1:1 into the CCRT arm or the CCRT + AC arm. The inclusion criteria were patients with age between 18 and 70 years old, histology-confirmed NPC, stage T2N0M0-T4N2M0 (AJCC 7th edition), Eastern Cooperative Oncology Group performance status 0–1, adequate bone marrow function, and serum creatinine clearance ≥ 30 mL/min. Exclusion criteria were N3 or metastatic disease.

The treatment was according to CCRT + AC arm in the prospective study [19]. The radiation techniques were ranged from conventional radiotherapy to intensity-modulated radiation therapy (IMRT) with the radiation dose 69.96–70 Gy, 59.4–60 Gy and 50–54 Gy to the gross tumor and the involved neck node, to the intermediate nodal area, and to low-risk nodal area, respectively. The treatment plan also included administration of weekly carboplatin 100 mg/m^2^ during radiotherapy for 6 cycles followed by 3 cycles of adjuvant carboplatin AUC 5 and fluorouracil at 1,000 mg/m^2^ intravenous infusion for 4 days every 28 days. This chemotherapy regimen was based on our previously established chemotherapy approach [20]. No patients underwent induction chemotherapy in this study.

Patient characteristics, including age, sex, and cancer staging, were collected. Complete blood count (CBC) was obtained from the three timepoints: at the first day of radiotherapy before starting CCRT (pre-CCRT), at the final week of CCRT (end-CCRT), and at the date of starting the last cycle of adjuvant chemotherapy (end-AC). NLR was calculated at each timepoint by dividing the absolute number of neutrophil counts by the absolute of lymphocyte counts from the CBC [5].

Pre-CCRT NLRs were divided into “low” and “high” NLR groups by the median NLR. End-CCRT and end-AC ΔNLR were categorized into “increased” and “decreased” groups based on NLR at these two timepoints relative to the value at pre-CCRT.

Patient was evaluated by endoscopy after treatment every 3 months for the first 2 years, then every 6 months afterward. Additionally, neck computed tomography or magnetic resonance imaging was conducted every 6 months for the first 2 years and annually thereafter. Overall survival was defined as the time between the treatment start date and the date of death or censorship at the date of the last visit. Disease-free survival was defined as the duration between treatment start date and the date of recurrence, death, or censoring.

This study was approved by the ethical committee of the Faculty of Medicine at Chiang Mai University with approval number 226/2022 and was conducted in accordance with the Declaration of Helsinki. The data was collected from the medical records between July 1 and September 30, 2022, after approval from the ethics committee, using a study number without the patient’s ID, HN, or name in order to protect patient confidentiality.

### Statistical analysis

Binary logistic regression was used to investigate the association between patient characteristics (i.e., sex, age, and cancer stage) and NLR which included NLR at pre-CCRT (low and high) and ΔNLR at end-CCRT and end-AC from pre-CCRT (increased and decreased). Kaplan-Meier analysis was used to determine 3-year mortality rates, and log-rank testing were used to compare overall survival according to sex, age, cancer stage, NLR at pre-CCRT, end-CCRT ΔNLR, and end-AC ΔNLR. Factors associated with OS and DFS were identified by using Cox proportional hazard regression models. NLR between the three treatment visits (i.e., pre-CCRT, end-CCRT, and end-AC) were compared using the Friedman test, with the application of Wilcoxon matched-pairs signed-ranks test to determine pairwise significance among these treatment visits. All data analyses were performed using Stata version 15.

## Results

Out of the 82 patients within the CCRT+AC arm, data was accessible for 81 patients. Among this cohort, there were 48 (59%) men and 33 (41%) women. The majority of patients, 69 (85%), exhibited an ECOG performance status score of 0, while the remaining 15% had a score of 1. The median age was 51 years (interquartile range [IQR]: 44–58). Most of the patients (47%) were diagnosed with stage III. The pathology indicated dedifferentiated non-keratinizing carcinoma in 4 patients (5%) and undifferentiated non-keratinizing carcinoma in 77 patients (95%). Median NLR at pre-CCRT, end-CCRT and end-AC were 2.47 (IQR 1.80–3.89), 6.29 (3.54–12.19), and 3.77 (2.54–5.78), respectively. Forty-one patients (51%) were defined as a high NLR at pre-CCRT. In addition, 81% patients had an increased ΔNLR in end-CCRT, and 74% had an increased ΔNLR in end-AC. The relationship between characteristics and NLR at pre-CCRT are reported in Table 1. Adjusted for sex and age, patients who had stage IVA at pre-CCRT were more likely to have high NLR at pre-CCRT than patients who had stage II at pre-CCRT (adjusted odds ratio [aOR] = 5.72, 95% confidence interval [95% CI]: 1.45–22.55).

**Table 1:** Relationship between characteristics and NLR at pre-CCRT.

Variables		Low NLR	High NLR				
		n/N (%)	n/N (%)	OR	aOR	95% CI of aOR	*P*
Sex							0.191
	Female (ref.)	15/33 (45)	18/33 (55)	1.00	1.00		
	Male	25/48 (52)	23/48 (48)	0.77	0.52	0.19–1.39	
Age (years)							0.092
	> 51 (ref.)	17/40 (42)	23/40 (58)	1.00	1.00		
	≤ 51	23/41 (56)	18/41 (44)	0.58	0.43	0.16–1.15	
Cancer stage							0.026
	Stage II (ref.)	13/21 (62)	8/21 (38)	1.00	1.00		
	Stage III	21/38 (55)	17/38 (45)	1.32	1.27	0.42–3.88	
	Stage IVA	6/22 (27)	16/22 (73)	4.33	5.72	1.45–22.55	

Abbreviations: ref., reference group; OR, odds ratio; aOR, adjusted odds ratio; 95%CI, 95% confidence interval.

Table 2 reports the association between characteristics at baseline and end-CCRT ΔNLR and end-AC ΔNLR. Adjusted for age and cancer stage at pre-CCRT, male was more likely to be an increased end-CCRT ΔNLR than female (aOR = 4.80, 95% CI: 1.36–16.95, *P* = 0.015). In addition, male and age ≤ 51 years were more likely to be an increased NLR at end-AC compared to their respective comparison group.

**Table 2:** Relationship between characteristics and end-CCRT ΔNLR and end-AC ΔNLR.

Variables		Increased ΔNLR at end-CCRT					Increased ΔNLR at end-AC				
		n (%)	OR	aOR	95% CI of aOR	*P*	n (%)	OR	aOR	95% CI of aOR	*P*
Sex						0.015					0.010
	Female (ref.)	23/33 (70)	1.00	1.00			20 (61)	1.00	1.00		
	Male	43/48 (90)	3.74	4.80	1.36–16.95		40 (83)	3.25	4.45	1.43–13.79	
Age (years)						0.213					0.032
	> 51 (ref.)	31/40 (78)	1.00	1.00			26 (65)	1.00	1.00		
	≤ 51	35/41 (85)	1.69	2.17	0.64–7.34		34 (83)	2.62	3.45	1.11–10.75	
Cancer stage						0.778					0.702
	Stage II (ref.)	13/21 (62)	1.00	1.00			17 (81)	1.00	1.00		
	Stage III	31/38 (82)	0.74	0.76	0.16–3.51		27 (71)	0.58	0.59	0.15–2.33	
	Stage IVA	17/22 (77)	0.57	0.38	0.07–2.08		16 (73)	0.63	0.42	0.09–2.03	

Abbreviations: ref., reference group; OR, odds ratio; aOR, adjusted odds ratio; 95%CI, 95% confidence interval

Table 3 reports the medians for NLR at pre-CCRT compared with end-CCRT and end-AC for all patients. Overall, median NLRs at end-CCRT and end-AC were higher than those at pre-CCRT (6.29 at end-CCRT and 3.77 at end-AC compared to 2.47 at pre-CCRT; *P* = 0.043), and the differences of NLRs according to pairwise test among these treatment visits were statistically significant (*P* <0.001).

**Table 3:** Median and interquartile range of NLR at pre-CCRT, end-CCRT and end-AC (n = 81).

Treatment visit		NLR		
		Median	interquartile range	*P*
				0.043^a^
	Pre-CCRT	2.47	1.80 to 3.89	
	End-CCRT	6.29	3.54 to 12.19	
	End-AC	3.77	2.54 to 5.78	
Pairwise comparison between treatment visits				
	Pre-CCRT vs. End-CCRT			<0.001^b^
	Pre-CCRT vs. End-AC			<0.001^b^
	End-CCRT vs. End-AC			<0.001^b^

Abbreviations: NLR, neutrophil to lymphocyte ratio; CCRT, concurrent chemoradiotherapy; AC, adjuvant chemotherapy.

^a^ *P*-value from Friedman test

^b^ *P*-value from Wilcoxon matched-pairs signed-ranks test

**Fig 1:**
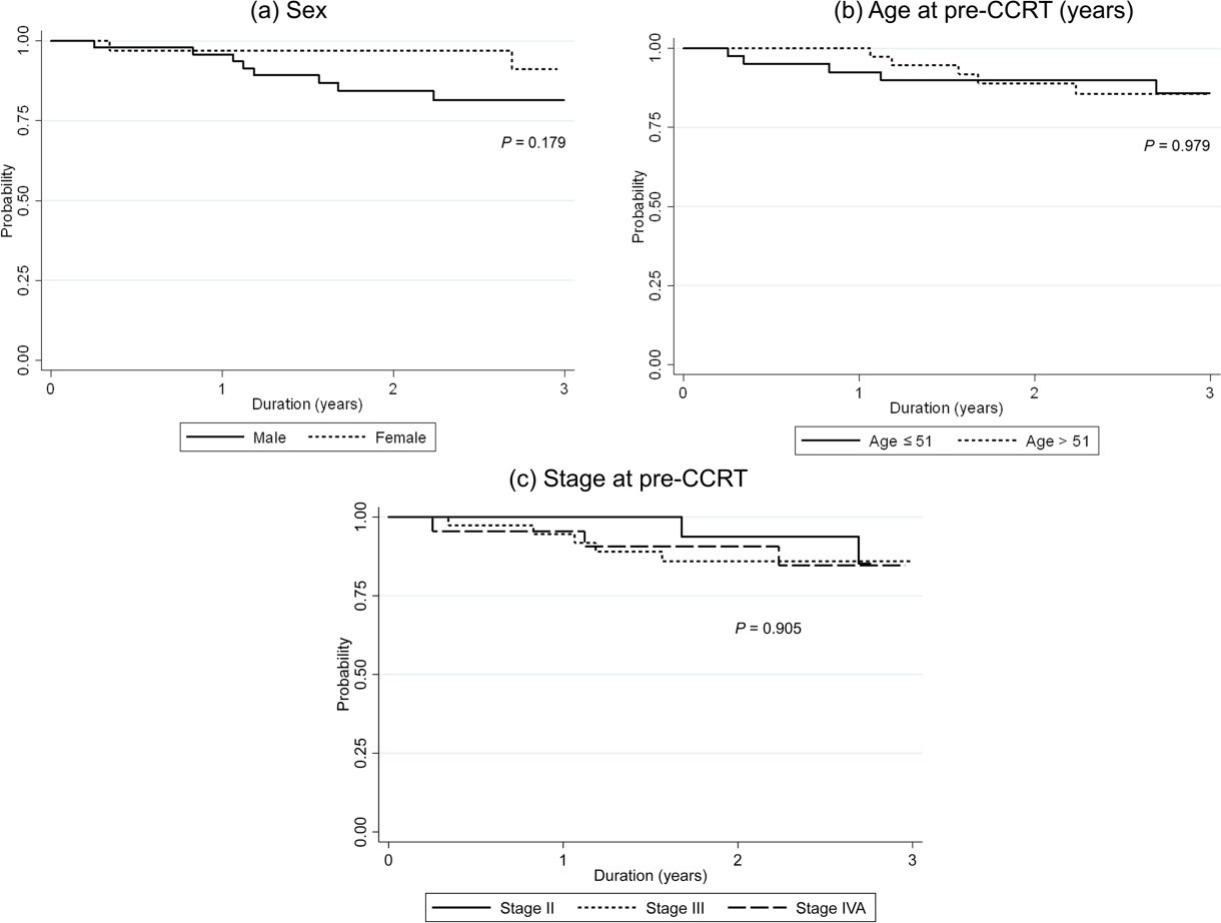
Three-year overall survival according to patient characteristics (a) sex, (b) age at pre-CCRT and (c) cancer stage at pre-CCRT. CCRT, concurrent chemoradiotherapy.

**Fig 2:**
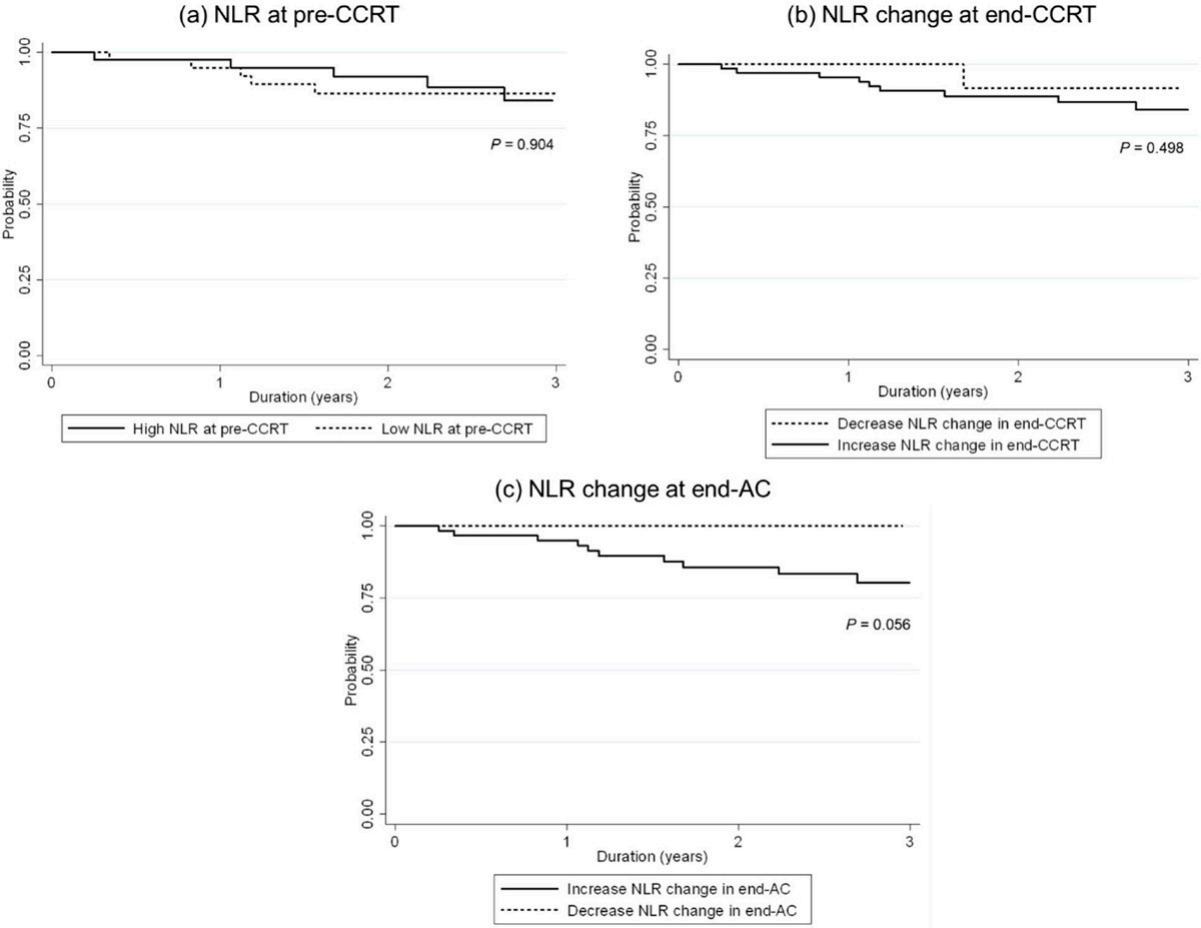
Three-year overall survival in subgroup with NLR and ALC at pre-CCRT and NLR and ALC change. (a) High and low NLR at pre-CCRT, (b) end-CCRT ΔNLR, and (d) end-AC ΔNLR. NLR, neutrophil to lymphocyte ratio; CCRT, concurrent chemoradiotherapy; AC, adjuvant chemotherapy.

With the median follow-up time of 2.58 years (IQR: 1.61 to 3.13), 3-year OS was 85.25% (95% confidence interval [CI]: 73.92% - 91.92%). The separate analytical results for patient characteristics at pre-CCRT (i.e., sex, age, and cancer stage) are in Fig 1A–1C, respectively. Kaplan-Meier plots of OS compared by sex, age and stage are shown in Fig 1. The results in Table 4 show there was no significant OS difference between their characteristics. Kaplan-Meier plot of OS in subgroup with high NLR compared to low NLR at pre-CCRT are shown in Fig 2A. There was no significant difference in OS between high NLR compared to low NLR at pre-CCRT, with a hazard ratio (HR) of 0.95 (*P* = 0.940). The separate analytical results for end-CCRT ΔNLR show in Fig 2B. There was no significant difference in OS between an increased and decreased end-CCRT ΔNLR (HR = 2.02, 95% CI: 0.26–15.91, *P* = 0.506). Fig 2C show the difference in OS between an increased and decreased end-AC ΔNLR were not significant (Log-rank test, *P* = 0.056).

**Table 4:** Factor associated with overall survival.

Variables		n/N	(%)	Cox regression analysis		
				HR	95% CI of HR	*P*
Sex						0.196
	Female (ref.)	2/33	(6)	1.00	–	
	Male	8/48	(17)	2.78	0.59–13.10	
Age						0.979
	> 51 (ref.)	5/40	(13)	1.00	–	
	≤ 51	5/41	(12)	1.02	0.29–3.52	
Stage						0.906
	Stage II (ref.)	2/21	(10)	1.00	–	
	Stage III	5/38	(13)	1.41	0.27–7.26	
	Stage IVA	3/22	(14)	1.44	0.24–8.63	
NLR at pre-CCRT						0.940
	Low (ref.)	5/40	(13)	1.00	–	
	High	5/41	(12)	0.95	0.28–2.30	
ΔNLR at end-CCRT from pre-CCRT						0.506
	Decrease (ref.)	1/15	(7)	1.00	-	
	Increase	9/66	(14)	2.02	0.26–15.91	
ΔNLR at end-CCRT from pre-CCRT						NA
	Decrease (ref.)	0/21	(0)	-	-	
	Increase	10/60	(17)	-	-	

Abbreviations: ref., reference group; HR, hazard ratio; aHR, adjusted hazard ratio; 95%CI, 95% confidence interval; NA, not applicable; NLR, neutrophil to lymphocyte ratio; CCRT, concurrent chemoradiotherapy; AC, adjuvant chemotherapy.

**Fig 3:**
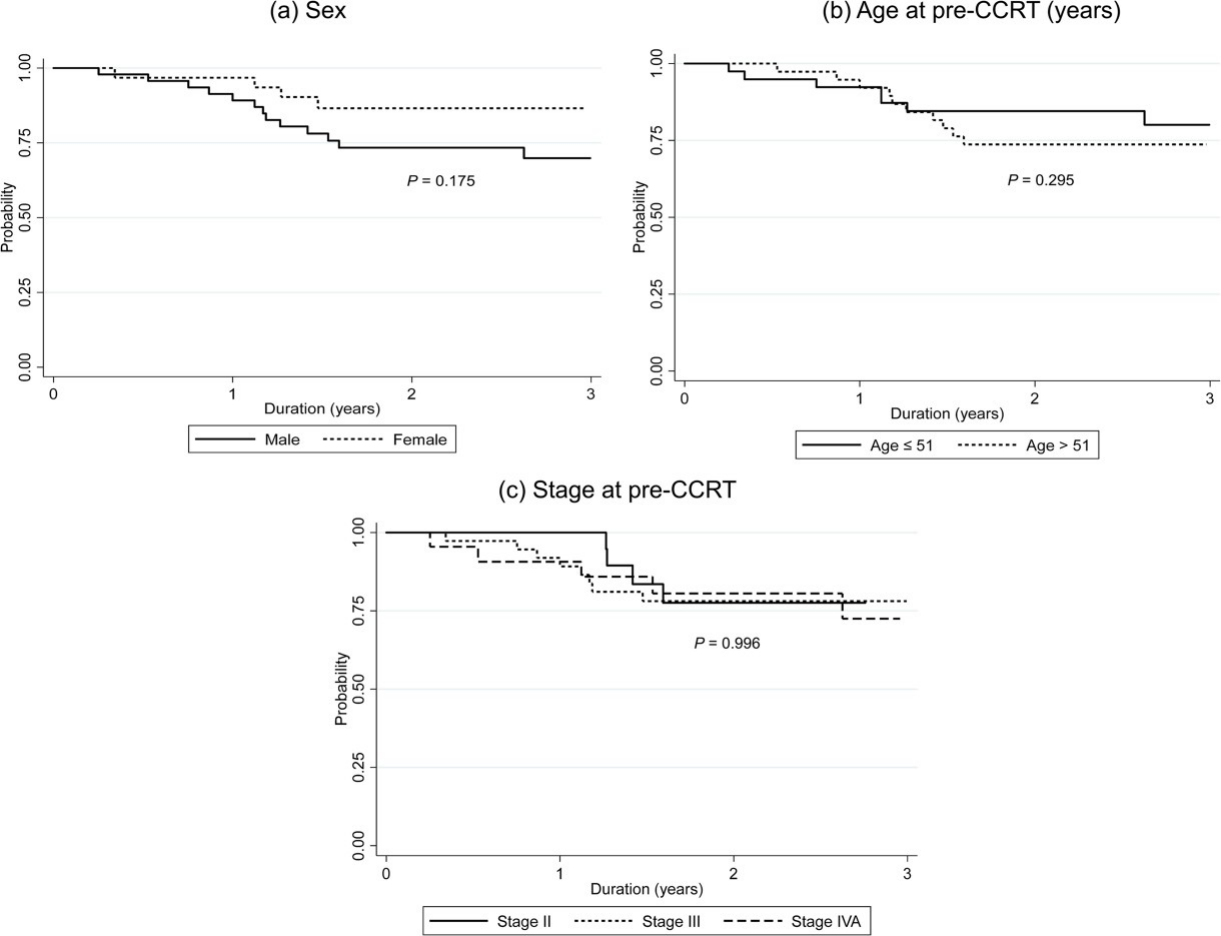
Three-year disease-free survival according to patient characteristics (a) sex, (b) age at pre-CCRT and (c) cancer stage at pre-CCRT. CCRT, concurrent chemoradiotherapy.

**Fig 4:**
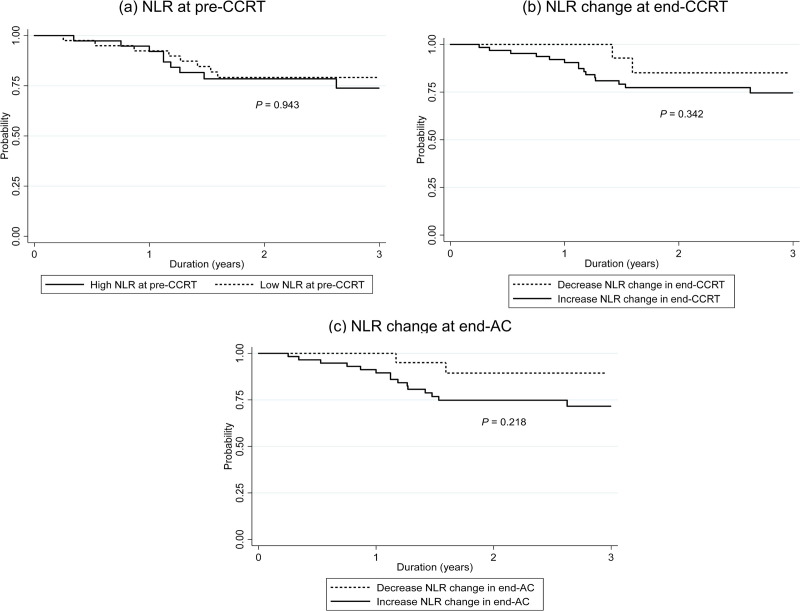
Three-year disease-free survival in subgroup with NLR and ALC at pre-CCRT and NLR and ALC change. (a) High and low NLR at pre-CCRT, (b) end-CCRT ΔNLR, and (d) end-AC ΔNLR. NLR, neutrophil to lymphocyte ratio; CCRT, concurrent chemoradiotherapy; AC, adjuvant chemotherapy.

Median DFS was 2.48 years (IQR: 1.38 to 3.11) and 3-year DFS was 76.39% (95% CI: 64.48% - 84.76%). Kaplan-Meier plots of DFS compared by sex, age and stage are shown in Fig 3. The results in Table 5 show there was no significant DFS difference between their characteristics. Kaplan-Meier plot of DFS in subgroup with high NLR compared to low NLR at pre-CCRT are shown in Fig 4A. There was no significant difference in DFS between high NLR compared to low NLR at pre-CCRT, with a hazard ratio (HR) of 1.03 (*P* = 0.943). The separate analytical results for end-CCRT ΔNLR and end-AC ΔNLR show in Fig 4B. There were no significant differences in DFS between groups with increased and decreased end-CCRT ΔNLR (HR = 2.01, 95% CI: 0.46–8.74, *P* = 0.325) or groups with increased and decreased end-AC ΔNLR (HR = 2.13, 95% CI: 0.62–7.34, *P* = 0.229).

**Table 5:** Factor associated with disease-free survival.

Variables		n/N (%)	Cox regression analysis		
			HR	95% CI of HR	*P*
Sex					0.184
	Female (ref.)	5/33 (15)	1.00	–	
	Male	14/48 (29)	2.00	0.72–5.55	
Age					0.979
	> 51 (ref.)	12/40 (30)	1.00	–	
	≤ 51	7/41 (17)	0.61	0.24–1.55	
Stage					0.906
	Stage II (ref.)	5/21 (24)	1.00	–	
	Stage III	9/38 (24)	1.05	0.35–3.16	
	Stage IVA	5/22 (23)	1.03	0.30–3.57	
NLR at pre-CCRT					0.943
	Low (ref.)	9/40 (23)	1.00	–	
	High	10/41 (24)	1.03	0.42–2.55	
ΔNLR at end-CCRT from pre-CCRT					0.352
	Decrease (ref.)	2/15 (13)	1.00	-	
	Increase	17/66 (26)	2.01	0.46–8.74	
ΔNLR at end-CCRT from pre-CCRT					0.229
	Decrease (ref.)	3/21 (14)	1.00	-	
	Increase	16/60 (27)	2.13	0.62–7.34	

Abbreviations: ref., reference group; HR, hazard ratio; aHR, adjusted hazard ratio; 95%CI, 95% confidence interval; NA, not applicable; NLR, neutrophil to lymphocyte ratio; CCRT, concurrent chemoradiotherapy; AC, adjuvant chemotherapy.

## Discussion

We evaluated baseline NLR, its changes through the treatments, and the impact of both parameters on the OS and DFS of NPC. Our results demonstrated that median NLRs statistically significant increased throughout CCRT and then decreased during adjuvant chemotherapy but remained higher than the value at pre-CCRT. These findings correlated with our previous study [9] that showed significant increase in NLR during CCRT. Many studies have also demonstrated that a higher NLR is correlated with more severe acute adverse effects [21, 22]. Our results implied that NPC treatments increased systemic inflammation and combination of RT and chemotherapy had a greater effect on the inflammatory process than chemotherapy alone. Since acute side effects such as oral mucositis and esophagitis become more severe with higher cumulative radiotherapy doses throughout CCRT [23]; accordingly, the inflammation markers increase over the course of CCRT [24, 25].

In addition, we evaluated the factors that were associated with high baseline NLR and increased ΔNLR during treatments. Our study showed high baseline NLR was associated with advanced cancer stage, but not with age and sex. This finding is consistent with the findings of Lu et al. [26] and Chua et al. [27] who found higher NLR in more advanced T and N stages but demonstrated no correlation with age or sex. Furthermore, Chua et al. [27] also demonstrated that higher pre-treatment EBV DNA titers linked to higher NLR. During the treatments, we found that increased end-CCRT ΔNLR was related to male gender, and increased end-AC ΔNLR was related to male gender and age ≤51 years. However, neither end-CCRT nor end-AC ΔNLRs were associated with cancer stage. As NLR correlates with oncological outcomes, we evaluated the correlation between these two variables and treatment outcomes. However, our findings revealed no differences in OS or DFS based on gender, age, or cancer stage, which was opposed to numerous studies that demonstrated a worse prognosis with older age [27–30], male gender [28], and more advanced stage [26, 27, 29, 30]. These may result from the limitations of our study due to the small number of patients and short follow-up period.

Our study showed no difference in OS or DFS between high and low pre-CCRT NLR, as well as between increased and decreased end-CCRT ΔNLR but a trend towards statistical significance in OS when comparing between increased and decreased end-AC ΔNLR. Even though our results were consistent with the results from Chua et al. [27] which showed no difference in OS or DFS between high- and low-NLR, numerous studies have shown the correlation between a higher NLR and its dynamic changes on inferior NPC outcomes [13, 14, 16, 26, 29, 31]. Lu et al. [26] showed NLR > 2.28 was associated with a lower 5-year OS and progression-free survival (PFS). Su et al. [31] and Yin et al. [13] conducted meta-analyses and discovered that high NLR was significantly correlated with poorer OS and PFS. Ou et al. showed a tendency for higher NLR in the cancer recurrence group and statistically significant worse OS in patients with post-RT NLR >7.05 [16]. Liu et al. showed increased NLR at the end of CCRT from the value before treatment >1.8 linked to poorer survival [29].

Our study is the first to evaluate NLR during NPC treatment with weekly carboplatin and adjuvant carboplatin and 5-FU. Unlike a lot of studies that primarily focus on NLR measurements before treatment initiation, we evaluated the effect of the dynamic change of NLR which is rarely used to predict outcomes in other studies. Furthermore, we have added depth to our study by uncovering the key factors that influence these NLR changes. It gathered data from a prospective study; consequently, our study had a small number of missing data. Nonetheless, our study had some limitations. Firstly, Due to the small sample size and short follow-up period, it is difficult to evaluate the effect of NLR on OS and DFS. Secondly, as a retrospective study based on one arm of a prospective study, there is a possibility of selective bias. Therefore, additional prospective studies with larger sample sizes are required to confirm the findings of this study.

In conclusion, our findings demonstrated that NLR increases during NPC treatment. More advanced NPC staging is associated with a higher baseline NLR. Increasing NLR from its baseline is associated with male gender at the end of CCRT as well as male gender and age ≤51 years at the end of AC. However, no relation between NLR and its dynamic change with either OS or DFS was demonstrated in our study.

## Supporting information

S1 Dataset (XLSX)
